# Phytoecdysteroids and Anabolic Effect of *Atriplex dimorphostegia*: UPLC-PDA-MS/MS Profiling, In Silico and In Vivo Models

**DOI:** 10.3390/plants12010206

**Published:** 2023-01-03

**Authors:** Eman Zaghloul, Heba Handousa, Abdel Nasser B. Singab, Mohey M. Elmazar, Iriny M. Ayoub, Noha Swilam

**Affiliations:** 1 Department of Pharmacognosy, Faculty of Pharmacy, The British University in Egypt (BUE), Cairo 11837, Egypt; 2 Department of Pharmaceutical Biology, Faculty of Pharmacy and Biotechnology, German University in Cairo (GUC), Cairo 11435, Egypt; 3 Department of Pharmacognosy, Faculty of Pharmacy, Ain Shams University, Abbassia, Cairo 11566, Egypt; 4 Center for Drug Discovery Research and Development, Ain Shams University, Cairo 11566, Egypt; 5 Department of Pharmacology, Faculty of Pharmacy, The British University in Egypt (BUE), Cairo 11837, Egypt

**Keywords:** *Atriplex dimorphostegia*, 20-hydroxyecdysone, LC–MS-MS, molecular docking, anabolic activity

## Abstract

*Atriplex dimorphostegia* (Saltbush) is an annual halophytic shrub that is widely distributed across various parts of Asia. The current study is the first to report the metabolites profile of the total ethanol extract of the aerial parts of *A. dimorphostegia* (TEAD), and its anabolic activity together with the isolated 20-hydroxyecdysone (20-HE) in orchidectomized male rats. TEAD was analyzed and standardized utilizing UPLC-PDA-ESI–MS/MS and UPLC-PDA-UV techniques, resulting in tentative identification of fifty compounds including polyphenols, steroids and triterpenoids. In addition, 20-HE was quantified, representing 26.79 μg/mg of the extract. Phytochemical investigation of TEAD resulted in the isolation of 20-HE from the ethyl acetate fraction (EFAD) and was identified by conventional spectroscopic methods of analysis. Furthermore, the anabolic effect of the isolated 20-HE and TEAD was then evaluated using in silico and in vivo models. Molecular docking experiments revealed in vitro selectivity of 20-HE towards estrogen receptors (ERs), specifically ERβ over ERα and androgenic receptor (AR). The anabolic efficacy of TEAD and 20-HE was studied in orchidectomized immature male Wistar rats using the weight of gastrocnemius and soleus muscles. The weights of ventral prostate and seminal vesicles were used as indicators for androgenic activity. Rats administered 20-HE and TEAD showed a significant increase (*p* = 0.0006 and *p* < 0.0001) in the net muscle mass compared to the negative control, while the group receiving TEAD showed the highest percentage among all groups at *p* < 0.0001. Histopathological investigation of skeletal muscle fibers showed normal morphological structures, and the group administered 20-HE showed an increase in cross sectional area of muscle fibers comparable to methandienone and testosterone groups at *p* > 0.99. *A. dimorphostegia* exhibited promising anabolic activity with minimal androgenic side effects.

## 1. Introduction

Since the discovery of testosterone in 1935, many derivatives have been synthesized to prolong its biological activity, rendering them orally active and developing products referred to as anabolic androgenic steroids (AAS) with maximum anabolic effects and minimum androgenic side effects [1]. Although AAS therapy has been associated with treating muscle contusion injury, hypogonadism and other chronic diseases such as cachexia, they are very common among recreational athletes [2,3]. Their prolonged administration, however, have been associated with a negative impact on fertility in both sexes related to the strong androgenic 5-α-dihydrotestosterone effect [3,4].

Interestingly, ecdysteroids, a class of hormones found in arthropods responsible for molting and reproduction, have shown anabolic effects in vertebrates and humans [5,6,7]. When compared to AAS, ecdysteroids do not bind to androgenic receptors (ARs). This is possibly because they affect the signal transduction pathway via membrane bound receptors such as estrogen receptors (ERs) and increase protein synthesis in skeletal muscles [6,8]. ERs are ligand-activated transcription factors belonging to the nuclear receptor family [9]. There are two ER subclasses, ER alpha (ERα) and ER beta (ERβ), with different tissue distribution and expression levels. ERs are not limited to reproductive organs; they are also involved in other biological activities in almost all mammalian tissues and organs [10] ERα is expressed primarily in reproductive tissues, while ERβ is mainly expressed in the gastrointestinal tract, vascular endothelial cells, and the prostate [11]. Both ERα and ERβ are expressed in skeletal muscles of mice and humans [10,12,13,14]. Accumulating evidence showed that selective activation of ERβ potently induces regeneration and fibrogenesis in toxin-injured muscle in female rats [15].

Phytoecdysteroids are the plant derived analogues of ecdysteroids, possessing a 14-α-hydroxy-7-en-6-one chromophore and a 27–29 carbon skeleton [16] Ecdysteroids have been discovered in more than 100 plant families [17]. The compound 20-hydroxyecdysone (20-HE) was identified with a structure identical to that isolated from insects [18]. The pharmacological effects of ecdysteroids on mammals have been investigated by monitoring body weight, organ and muscle weight after intraperitoneal administration, and the most widely investigated ecdysteroid was 20-HE due to its low acute toxicity [8,19]. 

20-HE was isolated from several members of genus *Atriplex* belonging to the family Chenopodiaceae [20,21,22,23,24]. The *Atriplex* genus comprises about 300 species of herbs and shrubs that grow throughout subtropical areas of the world, with the majority growing in saline soils and hence the name saltbush [23]. Several species of *Atriplex* including *A. halimus, A. lindleyi, A. laciniata, A. confertifolia* and *A. leucoclada* were investigated for their antidiabetic, antioxidant, antiplasmodial, anticholinesterse, cytotoxic and antifungal effects [24,25,26,27,28,29]. However, according to the literature, a few studies of *Atriplex dimorphostegia* var. Kar. & Kir were reported [30,31]

*A. dimorphostegia* is an annual, 15–30 cm tall herb with grey-green, succulent leaves growing in various parts of Asia [32]. *A. dimorphostegia* aerial parts have not undergone comprehensive phytochemical studies. Considering the cited studies, nothing was found in the literature reporting the metabolic profile *of A. dimorphostegia* aerial parts responsible for its anabolic potential. Thus, the present study aimed at metabolites profiling and standardization of the ethanol extract of *A. dimorphostegia* aerial parts (TEAD) adopting UPLC-PDA and UPLC-PDA-ESI-MS/MS techniques. In addition, the isolation and structural elucidation of the anabolic ecdysteroid 20-HE from the ethyl acetate fraction (EFAD) of *A. dimorphostegia* is conducted for the first time. Molecular modeling experiments were conducted to support that estrogen receptor (ER) binding, rather than androgen receptor (AR) binding, mediates the anabolic effect of 20-HE. Moreover, in vivo assessment of the anabolic and androgenic activities of the TEAD and the isolated 20-HE was applied in orchidectomized male rats.

## 2. Results

### 2.1. Extraction and Fractionation of A. dimorphostegia

This study aimed to prepare a standardized ethanol extract of the aerial parts of *A. dimorphostegia* (TEAD). This included extraction, fractionation and standardization. TEAD was fractionated using solvents of increased polarity *n*-hexane followed by ethyl acetate. The ethyl acetate fraction (EFAD) was used to isolate a pure sample of 20-HE [33]. The *n*-hexane fraction was isolated first as a step in the purification and extraction of relatively non-polar components.

### 2.2. Identification of 20-HE Isolated from EFAD

**Figure 1:**
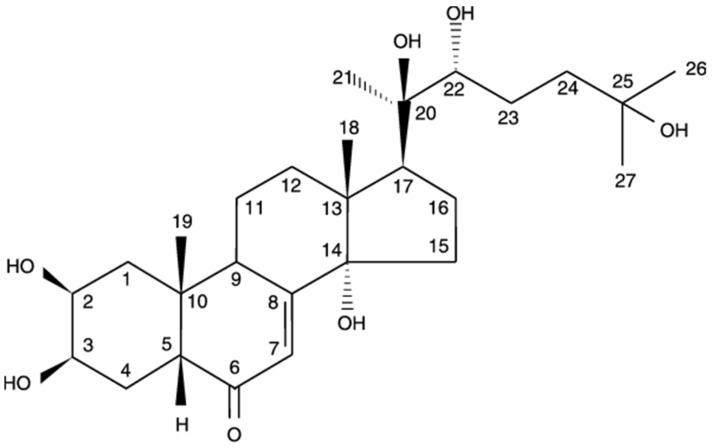
The structure of 20-HE.

Phytochemical investigation of the EFAD led to the isolation and identification of 20-HE as a white crystalline solid (Figure 1) (55 mg, mp. 237.5–239.5 °C) [34]. The purity of the compound was elucidated by HPLC and was found to be 99.5% [35]. The ^1^H NMR (400 MHz) spectrum recorded in DMSO d-6 showed five methyl groups at δH 0.74 (3H, s, H-18), 0.89 (3H, s, H-19), 1.22 (3H, s, H-21 and 3H, s, H-26) and 1.25 (3H, s, H-27), as well as a set of methylene group signals between δ H1.00 to δH 2.00, the ethylenic proton H-7 at δH 5.82 (1H, d, J = 2.2 Hz) and 3 CH-OH (δH 3.8), typical of the 20-HE skeleton. This was further confirmed by the ^13^C NMR spectrum which showed three O-bonded quaternary carbons (δC 71.3; 77.9; 85.2) and the presence of α, β-unsaturated ketone (δC 122.1; 168.0; 206.5) (Table 1) [36]. Finally, using the ESI-MS in positive mode showed a peak at *m*/*z* 481.243 corresponding to a [M+H]^+^ molecular ion. A molecular formula of C_27_H_44_O_7_ related to 20-HE was then deduced and confirmed according to the literature [36,37,38,39]. Further, the ^1^H and ^13^C NMR spectral data were closely related to those reported to the 5 β isomer of 20-HE [40]. This compound was used as a standard reference for the standardization of TEAD.

### 2.3. Characterization of Total Extract of A. dimorphostegia (TEAD) by UPLC-PDA-ESI-MS/MS

This is the first time to report the metabolic profile of TEAD utilizing Ultra-performance liquid chromatography (UPLC) coupled to photodiode array detection (PDA) and electrospray ionization (ESI) tandem mass spectrometry (MS) methods (UPLC-PDA-ESI-MS/MS) (Table 2)**.** Fifty compounds were tentatively identified, including phenolic acids and their derivatives, flavonoids, and their derivatives [41]. In addition, phytosterols, triterpenoids and steroids were also identified. 

Compound **1** was identified as caffeoyl-hexose-deoxyhexoside with a parent ion at *m*/*z* 487.01, showing MS^2^ ions at *m*/*z* 308 due to the loss of caffeic acid moiety, and at *m*/*z* 179 due to the expulsion of deoxyhexose and hexose moieties [42]. Compound **2** was identified with a [M−H]^−^ ion at *m*/*z* 451.12 showing fragmentation peaks at *m*/*z* 353, indicating the presence of caffeoyl-quinic acid or chlorogenic acid. The peak at *m*/*z* 191 is due to quinic acid and was identified as an unknown chlorogenic acid derivative [43,44]. Compound **3**, with a molecular ion peak at *m*/*z* 448.2 and exhibiting typical fragmentation patterns of C-glycosides, was assigned isoorientin [45,46]. Compound **4** with a molecular ion peak [M−H]^−^ at *m*/*z* 593.4 and fragment ions at *m*/*z* 353 [(M−H)−(120+120)]^−^ and at *m*/*z* 383 [(M−H)−(90+120)]^−^ indicates apigenin and two hexose moieties [45,47]. Compound **5** showed a parent ion [M−H]^−^ at *m*/*z* 417.01 and an MS^2^ fragment ion at *m*/*z* 255 ([M−H-glucose]^−^, liquiritigenin), and was assigned liquiritin [43,44]. Compound **6** was assigned ferulic acid with [M−H]^−^ at *m*/*z* 193.1 and a fragment ion peak at *m*/*z* 178 [M−H−15]^−^ due to the loss of a methyl group. The product ion at *m*/*z* 134 [M−H−15−44]^−^ is due to the loss of methyl and carboxyl groups [48]. Compound **7** presented with a base ion peak [M−H]^−^ at *m*/*z* 431.04 and MS^2^ ion at *m*/*z* 285 [M−H−146]^−^ due to the loss of a rhamnose moiety, and was assigned kaempferol rhamnoside [49]. Compound **8** has a [M−H]^−^ peak at *m*/*z* 153 characteristic of dihydroxybenzoic acid, while the ion at *m*/*z* 108 is due to the expulsion of the carboxyl moiety [50]. Compound **9** was identified as caffeic acid, showing a molecular ion peak [M−H]^−^ at *m*/*z* 179.01 and a fragment ion at *m*/*z* 135 corresponding to [M−H−CO_2_]^−^ [26,51]. Compound **10**, having precursor ion peak [M−H]^−^ at *m*/*z* 487.08, was identified as caffeoyl-hexose-deoxyhexoside. The fragment ion peak at *m*/*z* 308 is due to the loss of caffeic acid moiety and at *m*/*z* 179 is due to the expulsion of hexose and deoxyhexose [42]. Compound **11** exhibiting a molecular ion [M−H]^−^ at *m*/*z* 595.2 was assigned quercetin pentosyl hexoside. The MS^2^ fragment ion at *m*/*z* 463 [M−H−132]^−^ is due to the loss of pentose moiety and quercetin aglycone at *m*/*z* 301 [M−H−162−132]^−^ [26]. Compound **12** was suggested to be rosmarinic acid hexoside, showing a molecular ion [M−H]^−^ at *m*/*z* 521.1, and characteristic fragment ions at *m*/*z* 359 (rosmarinic acid) and at *m*/*z* 162 (hexoside moiety) [52]. Compound **13**, with a [M−H]^−^ ion at *m*/*z* 297 and MS^2^ fragments at *m*/*z* 282 and 267 due to loss of methyl and methoxyl groups, was identified as retusin methyl ether [53]. Compound **14** was identified as a caffeic acid ester derivative with a base ion peak at *m*/*z* 295. The fragment ion [M-116]^−^ *m*/*z* 179 is formed after the ester breakdown and the ion peak at *m*/*z* 135 [M-116-CO_2_]^−^ is characteristic of caffeic acid [53]. Compound **15** was identified as a ferulic acid ester derivative with [M−H]^−^ at *m*/*z* 309.53. The fragment ion at *m*/*z* 192 (ferulic acid) resulted from the ester breakdown and the loss of a residue [M−H−116]^−^. The fragment ion peak [M−H−116−CH_3_]^−^ at *m*/*z* 177 resulted from the loss of the methyl of the methoxy group [54]. Compounds **16**, **42** and **48**, with molecular ion peaks [M−H]^−^ at *m*/*z* 273, were assigned tetrahydroxyflavan (afzelechin), with the diagnostic product ion at *m*/*z* 179 due to the loss of the dihydroxybenzene moiety [48]. The data on compounds **17** and **43** were in agreement with the reported data on the fragmentation pattern of kaempferol aglycone [49,55]. Compounds **18** and **45** were tentatively identified as isorhamnetin, with [M−H]^−^ at *m*/*z* 315.2 and fragment ions at *m*/*z* 301 and 272 characteristic of isorhamnetin [26,56]. Compounds **19** and **46** with a molecular ion [M−H]^−^ at *m*/*z* 317 were tentatively identified as myricetin. The MS^2^ fragment ions at *m*/*z* 179 and 151 corresponds to the retrocyclization on the A–C ring and the loss of CO, respectively [26,56]. Compounds **20** and **47**, with base peak at *m*/*z* 301, matched the data reported for quercetin [26,57]. Compound **21** was identified as dihydroxybenzoyl hexose with a [M−H]^−^ ion at *m*/*z* 316.1, and a base peak at *m*/*z* 153 corresponding to dihydroxybenzoic acid [M−H−162]^−^ and *m*/*z* 108 due to the loss of the carboxyl group of benzoic acid [26,57]. Compound **22**, with molecular ion peak [M−H]^−^ at *m*/*z* 418.5, has a product ion at *m*/*z* 163 due to the loss of hydroxypalmatic acid moiety [M−H−256]^−^. The MS^2^ fragmentation pattern of *m*/*z* 163, 145, 119 and 93 is characteristic of the p-coumaroyl moiety. Thus, compound **22** was identified as coumaroyl-hydroxy-palmitic acid [48]. Compound **23**, having a [M−H]^−^ molecular ion at *m*/*z* 585, was identified as quercetin-galloyl-pentoside. In the MS^2^ spectrum, fragment ions at *m*/*z* 153 and *m*/*z* 301 correspond to the galloyl unit and quercetin aglycone units, respectively [58]. Compound **24** was identified as coumaroyl-hexose with the molecular ion [M−H]^−^ at *m*/*z* 325.65 and the MS^2^ ion at *m*/*z* 163 resulting from the loss of glucose or galactose [59]. Compound **25** showed an [M−H]^−^ peak at *m*/*z* 577 and a base peak at *m*/*z* 285 [M−H−292]^−^ owing to the loss of two hexoside moieties, and was suggested to be kaempferol-dideoxyhexoside (kaempferitrin) [49]. Compound **26** was identified as dicaffeoyl spermidine with a molecular ion peak [M−H]^−^ at *m*/*z* 468.2, a fragment ion at *m*/*z* 305 due to the loss of one caffeoyl moiety, and at *m*/*z* 145 corresponding to sperimidine with the loss of the two caffeoyl moeities [M−H−(162+162)]^−^ [51]. Compound **27** was identified as syringetin rutinoside with a molecular ion peak [M−H]^−^ at *m*/*z* 579.2 and a fragment ion peak at *m*/*z* 345 owing to the loss of the rutinoside moiety [M−H−(308)]^−^ [20,52]. Compound **28** showed an [M−H]^−^ peak at *m*/*z* 635.2 and a base peak at *m*/*z* 284 [M−H−351]^−^ owing to kaempferol, and was tentatively identified as acetylated kaempferol deoxyhexosyl hexoside [20,52]. Compound **29** with a [M−H]^−^ peak at *m*/*z* 593.17 was tentatively identified as kaempferol deoxyhexosyl hexoside. The base peak at *m*/*z* 285 [M−H−308]^−^ is due to the loss of the deoxyhexosyl moiety [49]. Compound **30** was identified as isorhamnetin hexoside, with a molecular ion peak at *m*/*z* 477.2 [M−H]^−^ and a base peak at *m*/*z* 315, attributed to the loss of hexose moiety producing the aglycone isorhamnetin [60]. Compound **31** was identified as quercetin deoxyhexoside with a base ion peak [M−H]^−^ at *m*/*z* 447, which produced a [M−H−146]^−^ ion at *m*/*z* 301 due to the loss of deoxyhexosyl moiety [62]. Compounds **32** and **38**, displayed base peaks at *m*/*z* 269 [M−H−162]^−^ in addition to fragment ions at *m*/*z* 341 [M−H−90]^−^ and 311 [M−H−120]^−^ and a molecular ion peak [M−H]^−^ at *m*/*z* 431.01, identical to apigenin-C-hexoside [63]. Compound **33** showed a molecular ion peak [M+H]^+^ at *m*/*z* 577 representing daucosterol (sitosterol-glucoside), with a fragment ion [M+H−162]^+^ at *m*/*z* 413 due to the loss of the hexose moiety corresponding to sitosterol, and the fragment ion at *m*/*z* 369 is due to the loss of glucosyl moiety followed by dehydration [64]. Compound **34**, showing a [M−H]^−^ ion at *m*/*z* 623.3 and a MS^2^ ion at *m*/*z* 315 (isorhamnetin) after the removal of the rutinose moiety, was identified as isorhamnetin deoxhexosyl hexoside [60] Compound **35** showed a [M−H]^−^ ion at *m*/*z* 461.07, and further fragmentation showed ions at *m*/*z* 285 and 175 corresponding to the aglycone kaempferol and glucouronide moiety. This compound was assigned kaempferol glucouronide [65]. Compound **36**, with an [M−H]^−^ at *m*/*z* 385, was identified as sinapic acid hexoside. The base peak at *m*/*z* 223 [M−H−162]^−^ was attributed to the loss of a hexosyl moeity [66]. Compound **37** showed a [M−H]^−^ molecular ion peak at *m*/*z* 623.2 and a base peak at *m*/*z* 315 (tamerexetin), and was tentatively identified as tamerexetin deoxyhexosyl hexoside [20]. Compound **39** was identified as caffeoyl-coumaroyl spermidine with a molecular ion peak [M−H]^−^ at *m*/*z* 452.4 [51]. Compound **40**, with a fragment peak ion [M−H_2_O+H]^+^ at *m*/*z* 397, was identified as β-sitosterol based on the fragments in the MS^2^ chromatogram [64,67]. Compound **41** had a [M−H]^−^ ion at *m*/*z* 519.5 showing MS^2^ peaks a at *m*/*z* 357 (pinoresinol), and the prominent ion at *m*/*z* 151 was due to the cleavage of the tetrahydrofuran ring [68]. Compound **44**, with a molecular ion peak [M−H]^−^ ion at *m*/*z* 237 and based on the spectral data, was identified as portulasoid (butoxyseptanoside) [36]. Compound **49** was identified as ursolic acid with a parent ion peak [M+H]^+^ at *m*/*z* 457 and a fragment ion peak [M−H_2_O-COOH+H]^+^ at *m*/*z* 393 [67,69] Compounds **50** and **52** both produced molecular ion peaks at *m*/*z* 427.2 and a base peak at *m*/*z* 218. They were identified as α-amyrin or β-amyrin, respectively [67,70]. Compound **51** with a molecular ion peak [M+H]^+^ at *m*/*z* 481 was tentatively identified as 20-hydroxyecdysone. The MS^2^ chromatogram showed sequential loss of water with a base ion peak M+H−2H_2_O]^+^ at *m*/*z* 445 and molecular ion peaks M+H−3H_2_O]^+^, M+H−4H_2_O]^+^ and [M+H−5H_2_O]^+^ at *m*/*z* 427, 409 and 391, respectively [36,71]. Compound **53** was identified as lupeol. The molecular ion peak [M+H]^+^ at *m*/*z* 427.5 showed a fragmentation of [M+H−H_2_O]^+^ at *m*/*z* 409 and at *m*/*z* 397 due to the loss of two methyl groups [36,71,72,73]. Compound **54** had a molecular ion peak [M+H]^+^ at *m*/*z* 413.2, showing fragment ions [M+H−H_2_O]^+^ and [M+H−2H]^+^ at *m*/*z* 395 and 411, respectively [73,74] Compound **55** was identified as olenoloic acid with a molecular ion peak [M+H]^+^ at *m*/*z* 457. The MS^2^ fragment ions were [M+H−H_2_O]^+^ at *m*/*z* 439, [M-COOH+ H]^+^ at *m*/*z* 411 and [M-COOH-H_2_O+H]^+^ at *m*/*z* 393 [67,75]. Compound **56** showed a peak [M−H]^−^ at *m*/*z* 657.2 and was identified as septanoecdysone [36].

### 2.4. Standardization of TEAD Using UPLC Analysis

**Figure 2:**
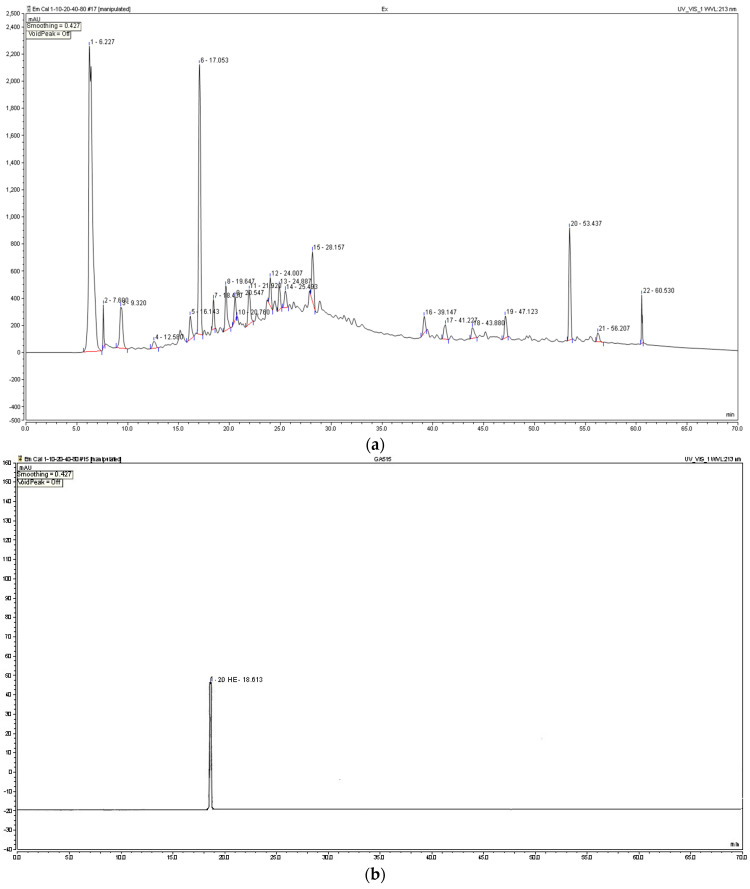

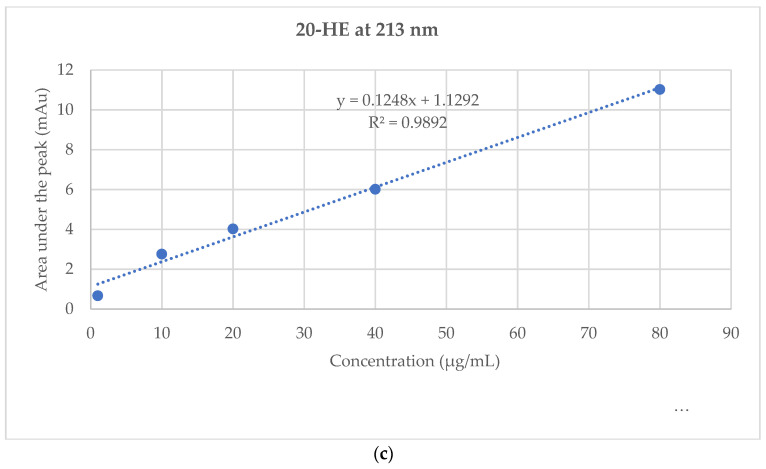
Standardization of TEAD: (**a**) HPLC chromatogram of TEAD at 213 nm. (**b**) UPLC chromatogram of the isolated 20-HE. (**c**) Calibration curve of the isolated 20-HE.

TEAD was analyzed using Ultra Performance Liquid Chromatography (UPLC). The PDA chromatogram of TEAD was recorded at 213 nm (Figure 2a) and showed an inset at Rt = 18.43 min, corresponding to 20-HE. A calibration curve of the isolated 20-HE was established on the same UPLC device with the same conditions and parameters. The calibration curve equation was established as y = 0.1248x + 1.1292 with a correlation coefficient of R² = 0.9892 (Figure 2b). Each mg of TEAD was shown to contain 26.79 ± 0.57 µg of 20-HE.

### 2.5. In Silico Modelling of Steroid Receptor Binding

**Figure 3:**
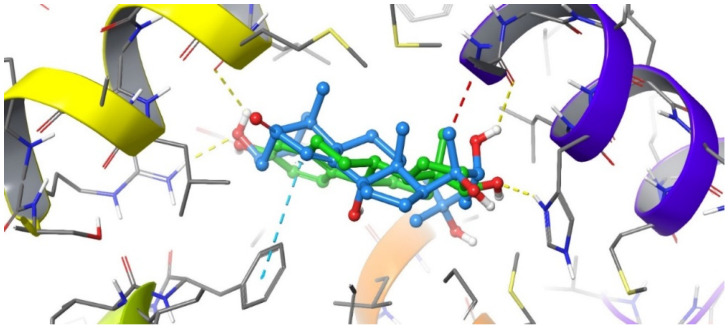
Overlay of the ligand 20-HE (cyan) and the co-crystallized ligand estradiol (green) in the binding cavity of ERβ. Hydrogen bonds are displayed with yellow dashed lines, while steric clashes are displayed in red dashed lines.

**Figure 4:**
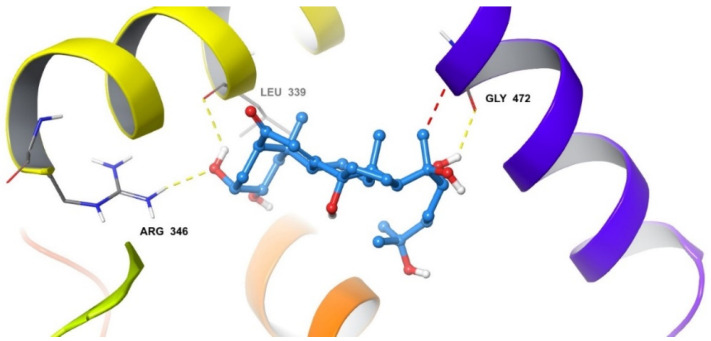
The binding poses of 20-HE in the binding site of ERβ. Hydrogen bonds are noted with Leu339, Arg346 and Gly472 (yellow dashed lines). A single clash at 2.75 Å is found with Gly472 (red dashed line).

The docked pose of 20-HE in the binding site of ERβ reveals a binding mode similar to that of the co-crystallized ligand, estradiol. The inhibitor occupies the hydrophobic central cavity of the binding pocket. The inhibitor is then anchored to the binding pocket via an array of hydrogen bonds. The cyclic end of the inhibitor is hydrogen bonded to the side chain of Arg346 and the backbone carbonyl of Leu339. Although the hydroxyl group of 20-HE occupies a virtually nearly identical position in space as the hydroxyl group of estradiol, it forms hydrogen bonds to different residues. The estradiol hydroxyl group is hydrogen bonded to the side chain of Glu305. Extending to the opposite side of the binding cavity, the aliphatic chain of 20-HE also contains a hydroxyl group that occupies a very similar position to that of the other hydroxyl group of estradiol. This hydroxyl group hydrogen bonds to the carbonyl of Gly472. Overall, the positioning of 20-HE is very similar to that of estradiol in the binding cavity of ERβ (Figure 3 and Figure 4).

**Figure 5:**
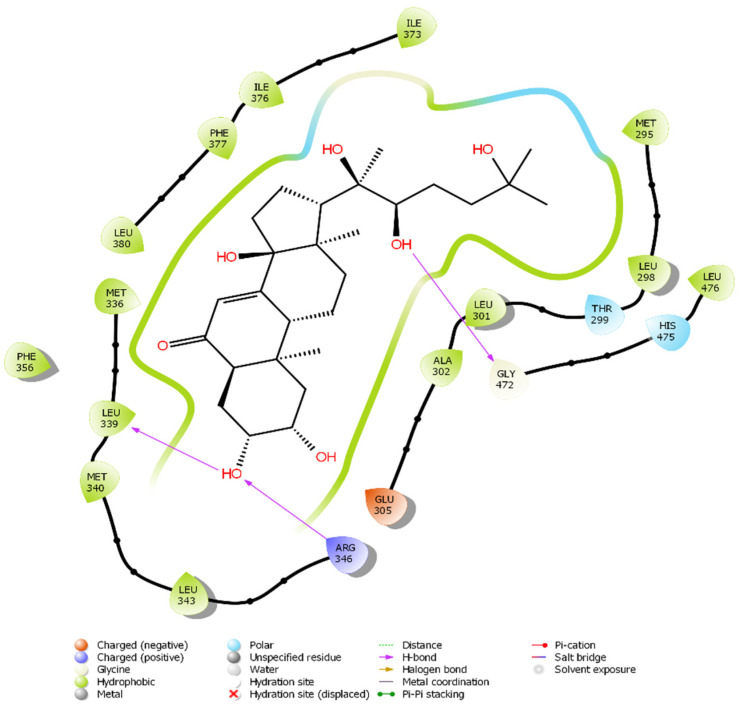
2D diagram of the binding of 20-HE to the binding site of ERβ.

**Figure 6:**
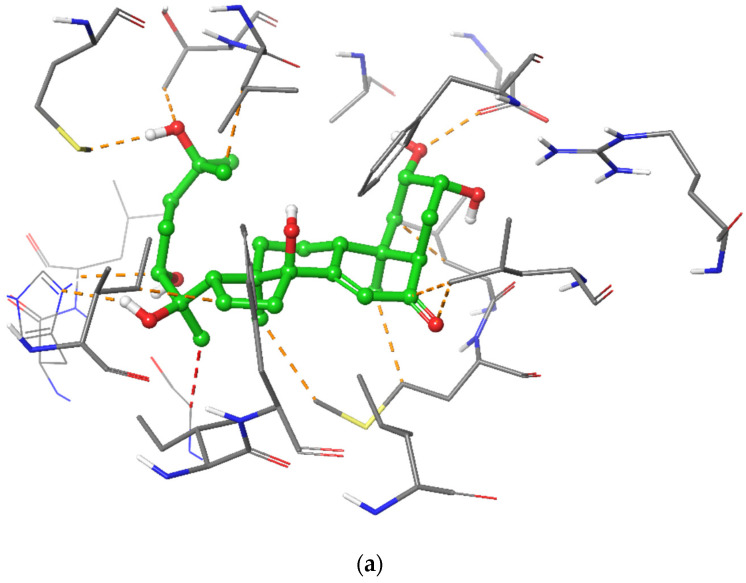

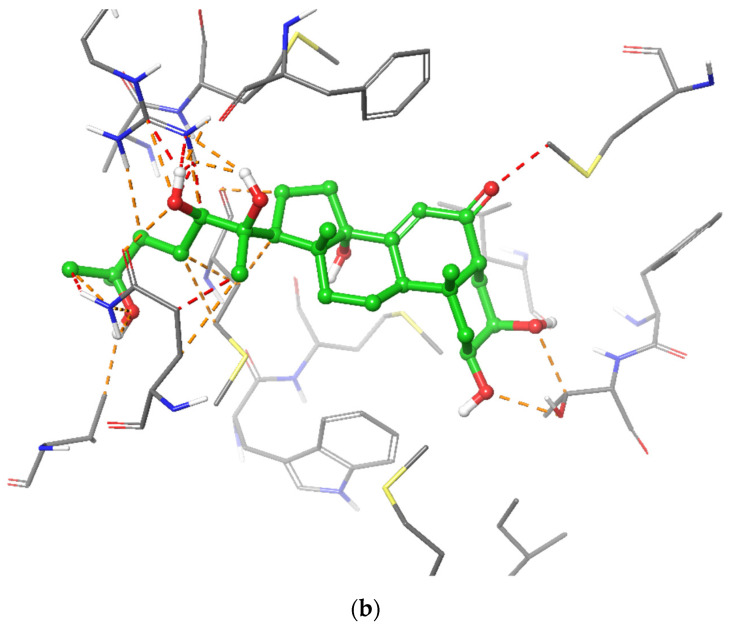
Clashing of 20-HE in the binding sites of ERα (**a**) and AR (**b**). Dashed lines in orange indicate unfavorable distances, while those in red indicate disallowed distances.

Looking at the binding modes of 20-HE in the cavity of ER*α* and AR immediately points out a critical determinant for selectivity towards ERβ (Figure 5). The cyclic end of 20-HE clashes with the side chain of Glu52. The aliphatic chain of the ligand clashes extensively with the side chains of His524 and Leu525. This clashing is governed by the secondary hydroxyl group and the terminal tertiary hydroxyl group (Figure 6). This suggests that despite the absence contribution of the extensive hydroxylation of the aliphatic chain of 20-HE to its binding to ERβ, the presence of these groups govern its selective binding towards ER*β* over ERα indirectly. Similarly, extensive clashing with the binding site of AR inhibits 20-HE binding to AR. The ketone group of the ligand clashes with the side chain of Met780. The aliphatic chain of the ligand clashes extensively with the side chains of Gln711 and Arg752. Similar to the case of ER*β*, the secondary hydroxyl group and the terminal methyl group of the ligand aliphatic chain are responsible for the ligand clashes, in addition to the methyl group of the C1 position of the aliphatic chain.

The C1 methyl group of the aliphatic chain of the ligand is of specific interest. It is responsible for additional clashing with the side chain of Gln711 of AR. However, it is also responsible for the only steric clash in the binding pose of 20-HE to ERβ with the carbonyl of Gly472 at 2.75 Å. Therefore, although this group potentially contributes to selectivity towards ER*β*, its elimination might be advantageous as it would increase the binding stability of the ligand to ER*β*. Its contribution in steric clashes with the side chain of both ERβ and AR suggests that the elimination of this would still conserve selectivity towards ERβ. Future medicinal chemistry studies can be directed towards the total synthesis of 20-HE analogues and the structure–activity relationship investigation of the effect of elimination of this methyl group on both the potency and selectivity of 20-HE.

In conclusion, 20-HE exhibits a binding pattern to ERβ similar to the co-crystallized ligand estradiol. The flexibility and poly-substitution of its aliphatic side chain mediates its selectivity towards ERβ via extensive clashing with the residue side chains within the binding site of ERα and AR. These steric clashes negatively influence the binding stability of 20-HE in the binding site of ERα and AR, and indirectly improve selectivity towards ERβ.

### 2.6. Evaluation of the Cytotoxic Activity of 20-HE and TEAD (SRB-U Assay)

**Figure 7:**
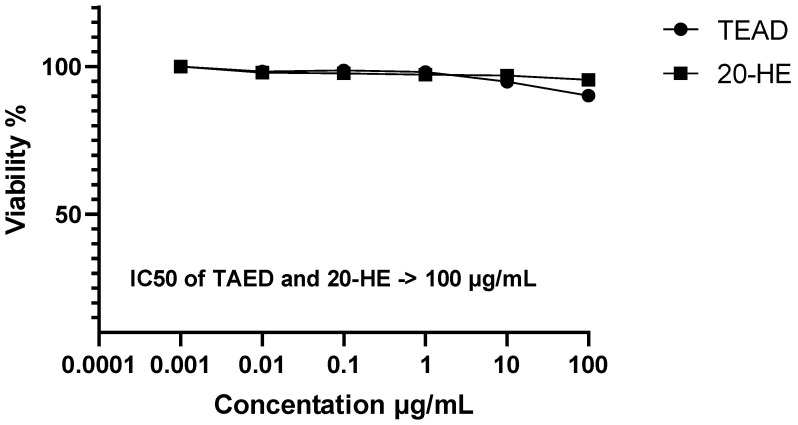
Cell viability and IC_50_ values of TEAD and 20-HE on skeletal muscle cells treated for 24 h.

Before assessing the anabolic activity of 20-HE and TEAD, their cytotoxicity was evaluated against human skeletal muscle cells (SkMC) using SRB-U assay for 24 h, human SkMC was subjected to 20-HE and TEAD at concentrations of 0.01, 0.1, 1, 10 and 100 µg/mL (Figure 7). 20-HE and TEAD exhibited no effect on cell viability at concentrations equal to or greater than 0.01 µg/mL with an IC_50_ of > 100 µg/mL.

### 2.7. In Vivo Anabolic Study

#### 2.7.1. Weight Gain and Mass of Muscle Bundle

**Figure 8:**
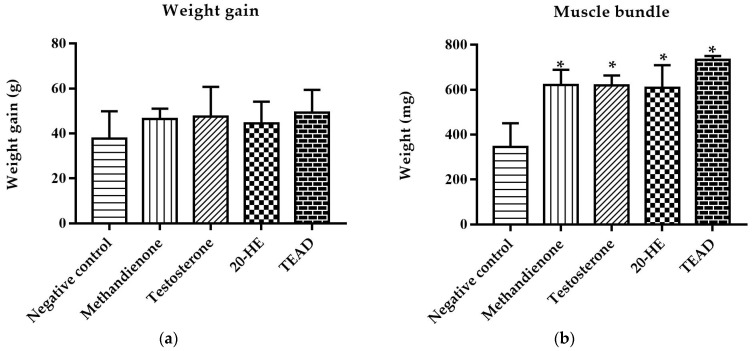
Weight gain of treated groups (**a**). No significant differences within groups, *p* = 0.54. Mass of gastrocnemius and soleus muscles of treated groups (**b**). *: Significantly different from the negative control group at *p* < 0.001.

After 10 days, there was no significant (*p* = 0.54) increase in total body weight among all groups. However, groups receiving methandienone and testosterone showed a significant (*p* < 0.0001) increase in the mass of muscle bundle compared to the negative control, both by 80%. Similarly, the administration of 20-HE and TEAD increased the net mass of muscles significantly (*p* = 0.0006 and *p* < 0.0001) compared to the negative control by 76% and 112%, respectively. There was no significant (*p* > 0.5) difference between 20-HE and TEAD (Figure 8).

#### 2.7.2. Weight of Secondary Sex Organs

**Figure 9:**
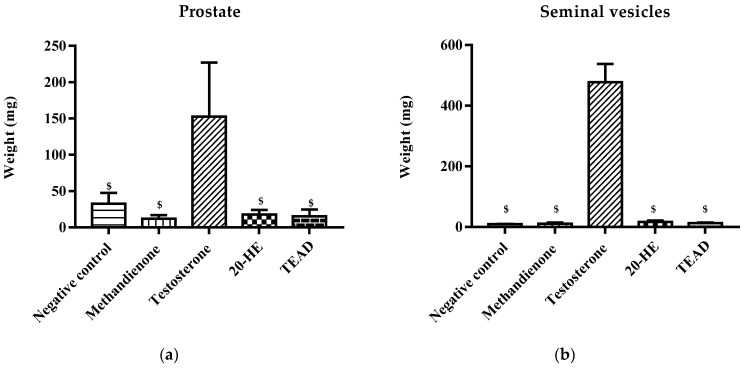
Mass of prostate (**a**) and seminal vesicles (**b**) of treated groups. ^$^: Significantly different from the testosterone group at *p* < 0.0001.

Group C receiving testosterone showed a significant (*p* < 0.0001) increase in the mass of prostate and seminal vesicles, a 14-fold increase in comparison with the negative control. Moreover, when comparing group C to groups receiving 20-HE and TEAD, the mass of prostate and seminal vesicles also increased significantly (*p* < 0.0001), by 17-folds (Figure 9).

#### 2.7.3. Anabolic to Androgenic Ratios

**Figure 10:**
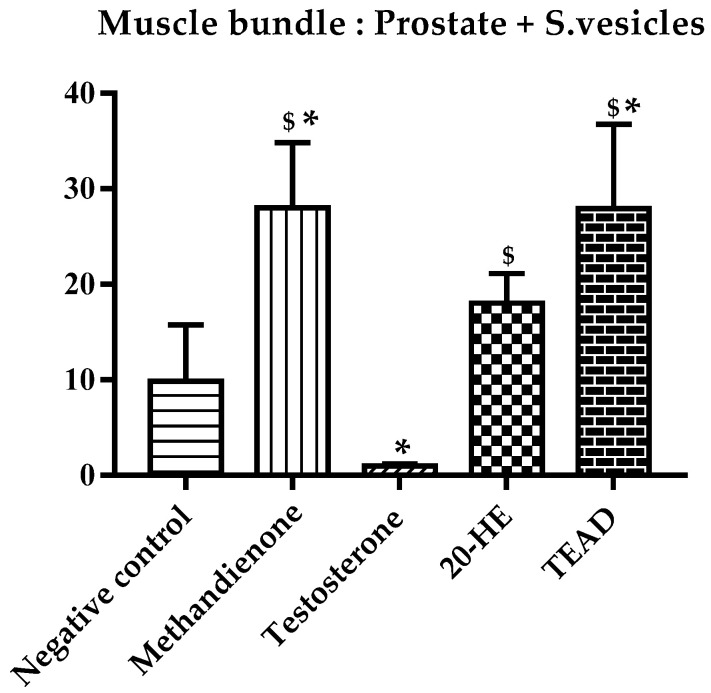
Average muscle bundle mass to secondary sex organs ratio. *: Significantly different from the negative control group at *p* < 0.001. ^$^: Significantly different from the testosterone group at *p* = 0.0011.

**Figure 11:**
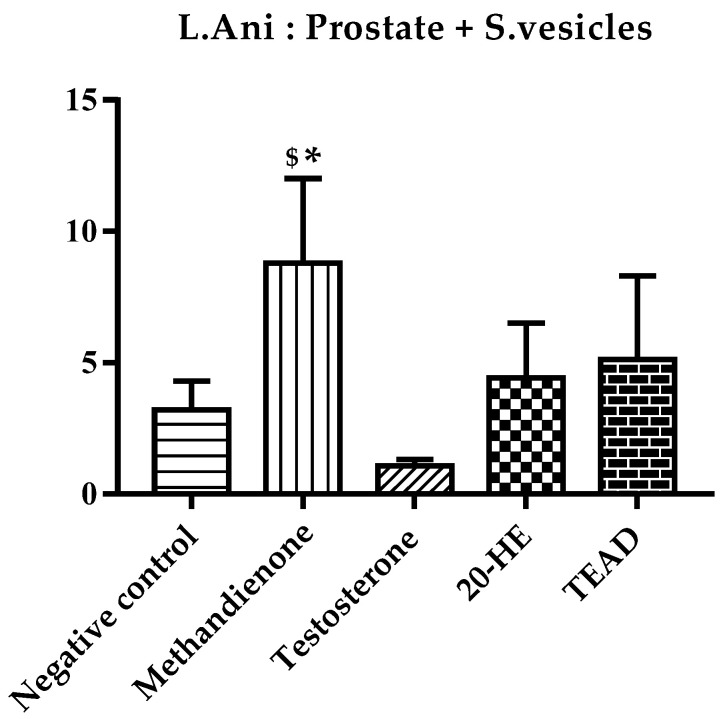
Average levator ani muscle mass to secondary sex organs ratio. *: Significantly different from the negative control group at *p* = 0.01. ^$^: Significantly different from the testosterone group at *p* = 0.0005.

The anabolic to androgenic ratio of each group was calculated first using the weight of the muscle bundle (Figure 10) and then using the weight of the levator ani muscle (Figure 11) against both the weights of the prostate and seminal vesicles. The group receiving methandienone had the highest anabolic ratios (27.92 and 8.79), showing a significant difference when compared to the negative control at *p* values of 0.001 and 0.01, respectively. The group receiving testosterone showed the lowest ratios (1.01 and 1.07), significantly different from the methandienone group at *p* values of <0.0001 and 0.0005, respectively.

The muscle bundle to prostate and seminal vesicles ratio showed a significant difference in the group receiving TEAD when compared to the negative control and testosterone groups at *p* values of 0.002 and <0.0001 respectively. There is no significant (*p* > 0.99) difference between the group receiving TEAD when compared to methandienone, and neither is there a significant difference when compared to 20-HE (*p* > 0.99). When comparing the group receiving 20-HE compared to the negative control, the difference is not significant, but the difference compared to testosterone is significant at *p* = 0.0036. Although the group receiving total extract showed a higher ratio when compared to 20-HE, the difference was not significant (*p* > 0.1).

#### 2.7.4. Muscle Mass Percentage

**Figure 12:**
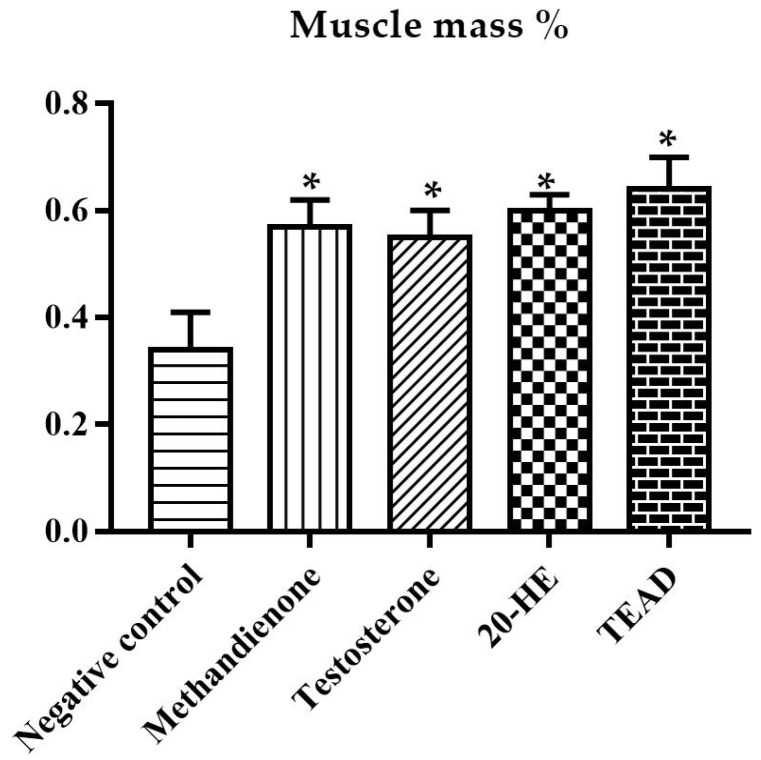
Muscle mass % of treated rats. *: Significantly different from the negative control group at *p* < 0.0001.

The muscle mass percentage was calculated using the mean mass of the muscle bundle of both right and left hindlimbs against the mean final body weight of rats in each group at the end of the study (Figure 12). The group receiving TEAD showed the highest percentage (0.64%) among all groups, two-fold compared to the negative control. However, the difference between the groups receiving TEAD and 20-HE was not significant. 

#### 2.7.5. Histopathological Examination with Hematoxylin and Eosin (H&E) Stain

**Figure 13:**
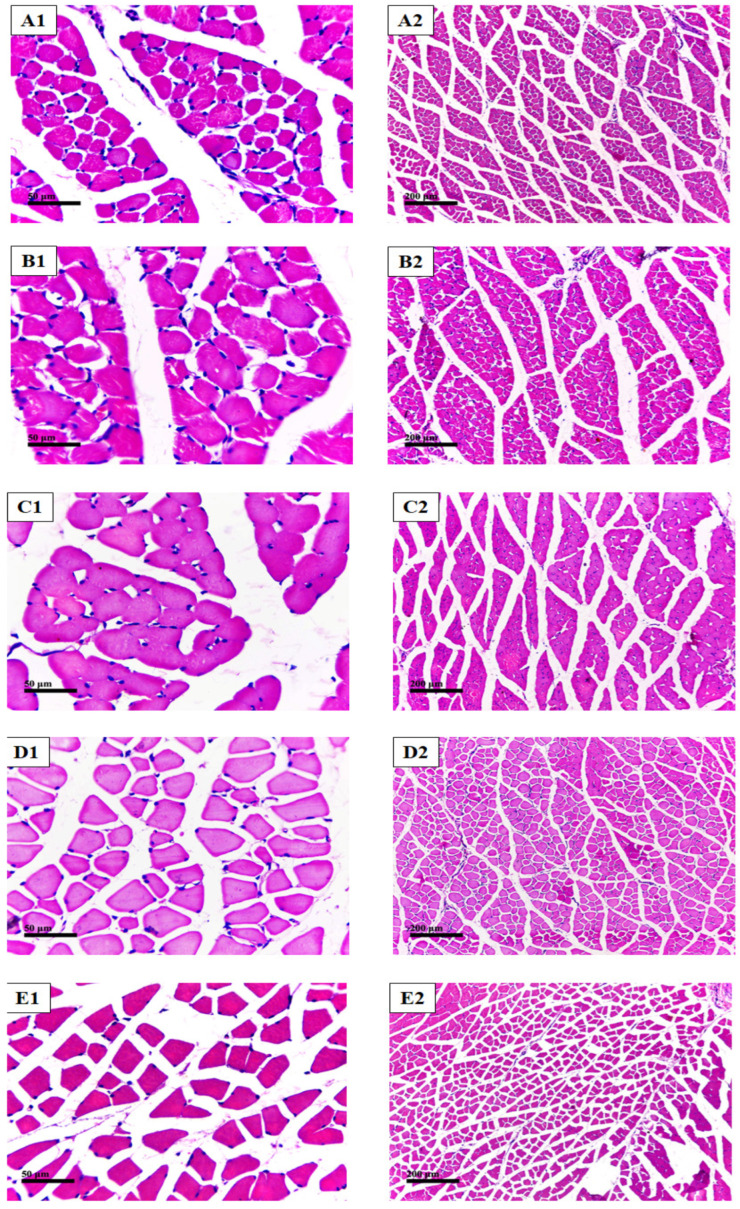
(**A1**,**A2**): sections from the negative control. (**B1**,**B2**), (**C1**,**C2**), (**D1**,**D2**), (**E1**,**E2**): sections from groups receiving methandienone, testosterone, 20-HE and TEAD, respectively.

All samples stained with H&E demonstrated almost normal morphological structures of skeletal muscle fibers, without abnormal subcellular alterations with normal intact intermuscular fine connective tissue, without abnormal inflammatory cell infiltrates and with normal vasculatures (Figure 13).

#### 2.7.6. Cross Sectional Area of Muscles

**Figure 14:**
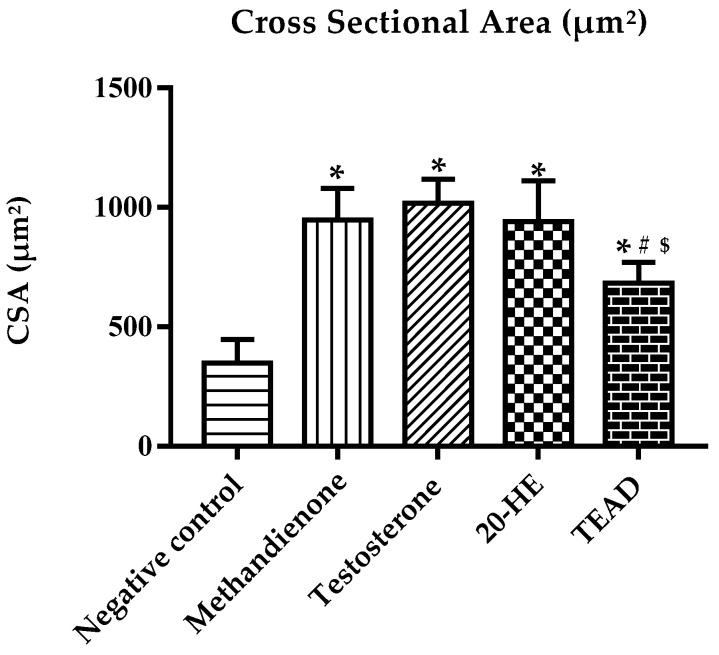
Cross sectional area of treated rats. *: Significantly different from the negative control group at *p* < 0.0001. ^$^: Significantly different from the testosterone group at *p* < 0.0001. ^#^: Significantly different from the methandienone group at *p* = 0.0004.

The size of muscles increased (*p* < 0.0001) in all groups compared to the negative control (Figure 14). While testosterone treatment showed a maximum increase in muscle fiber size (1017 ± 100 µm²), there was no significant (*p* = 0.67) difference when compared to 20-HE (941 ± 168 µm²). Similarly, no significant difference (*p* > 0.99) when comparing methandienone (946 ± 132 µm²) to 20-HE treatments. However, TEAD treatment (683 ± 86 µm²) showed significant difference when compared to testosterone (*p* < 0.0001) and methandienone (*p* = 0.0004).

#### 2.7.7. Number of Muscle Fibers

**Figure 15:**
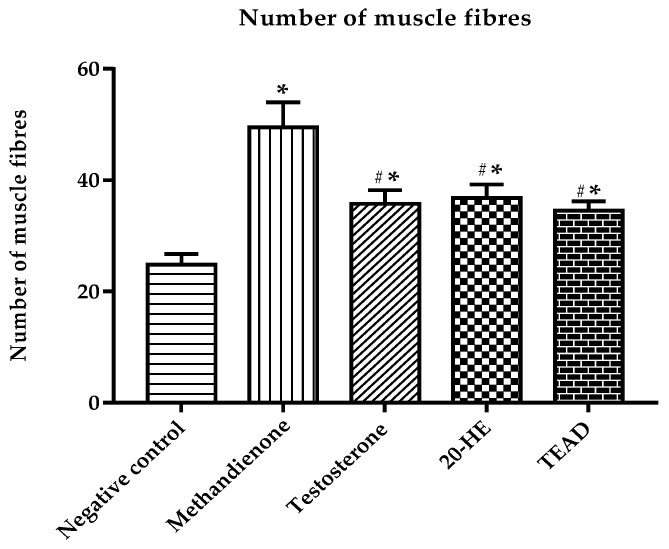
Number of muscle fibers. *: Significantly different from the negative control group at *p* < 0.0001. ^#^: Significantly different from the methandienone group at *p* < 0.0001.

The number of muscle fibers increased in all groups when compared to the negative control at *p* < 0.0001 (Figure 15). The group receiving methandienone showed the highest number of muscle fibers (49.5 ± 4.5), significantly higher than the groups receiving 20-HE and TEAD at *p* < 0.0001. There was no significant difference (*p* = 0.6) between the groups receiving 20-HE and TEAD. 

## 3. Discussion

The purpose of the current study was to evaluate the anabolic effect of *A. dimorphostegia*. TEAD was found to contain 20-HE (26.79 µg/mg), a phytoecdysteroid that was proven to have low acute toxicity [8] and an anabolic potency that have been well documented [6,7,19,76]. Several steroids have been employed in treating various muscle injuries and chronic diseases, as well as among athletes [2,3,16]. However, the negative impact of AAS on fertility in both sexes presents ecdysteroids as an alternative for AAS due to their anabolic potency with minimal androgenic side effects [8]. 

The UPLC-PDA-MS/MS profiling of TEAD highlighted the presence of a range of metabolites including polyphenols, phenolic acids, flavonoid glycosides and aglycones. Examples include coumaroyl hydroxy-palmitic acid, quercetin galloyl pentoside, acetylated kaempferol hexoxyl-rhamnoside and quercetin rhamnoside. In addition to 20-HE as the major identified compound, sterols, steroids and triterpenoids such as β-sitosterol, stigmasterol and β/α-amyrin were also identified. This comes in context with previous reports which refers to the occurrence of different types of polyphenols and steroids previously identified from different *Atriplex* species [36,43,45,49,50,67,73]. However, the current study is the first to report these compounds in *A. dimorphostegia*. 

Several studies reported that polyphenols such as apigenin quercetin and phenolic acids are highly effective in attenuating muscle atrophy and enhancing muscle health. Apigenin reduced oxidative stress and inhibited hyperactive autophagy and apoptosis, alleviating age-related skeletal muscle atrophy in rats [77,78]. Kanzaki et al. [79] reported that quercetin glycosides increased the wet weights of the quadratus femoris, gastrocnemius, tibialis anterior and soleus muscles in mice. While in the tibialis anterior and soleus muscles of rats, ferulic acid increased the production of mechano-growth factor and the antioxidant enzymes [80]. Moreover, Ommati et al. [81] proved that chlorogenic acid improved muscular strength in resistance-trained rats through improving mitochondrial activity and cellular energy metabolism. Phytosterols, such as β-sitosterol, according to Verma et al., by having an activity similar to that of 5-α-reductase, the enzyme that catalyzes the conversion of testosterone to 5-α-dihydrotestosterone, may be involved in the reported anabolic effect in the form of weight gain and muscle mass [82]. Moreover, ursolic acid was found to reduce muscle atrophy and stimulate muscle hypertrophy in mice by enhancing skeletal muscle insulin/IGF-I signaling and inhibiting atrophy-associated skeletal muscle mRNA expression [83].

To understand the practical in vitro selectivity pattern of 20-HE towards ERβ over ERα and the AR, molecular docking experiments were conducted using all three receptors. In these experiments, the protein data bank (PDB) entries 3OLL, 3UUD and 2AM9 were used for ERβ, ERα and AR, respectively [84,85,86]. These crystal structures were specifically selected as they are in ligand-bound states and therefore their conformations would allow ligand docking into the ligand-binding site. The docked poses of 20-HE revealed that the steric clashes negatively influence the binding stability of 20-HE in the binding sites of ERα and AR, and indirectly improve selectivity towards ERβ. These findings were in agreement with previous studies [8,87]

The anabolic efficacy of TEAD and the isolated 20-HE was evaluated using testosterone enanthate and methandienone as positive controls in orchidectomized immature male Wistar rats [6]. The groups administered 20-HE and TEAD showed a significant increase (*p* = 0.0006 and *p* < 0.0001) in the net mass of muscles compared to the negative control, while the group receiving the TEAD showed the highest percentage (0.64%) among all groups. Tóth et al. proved that 20-HE increased fiber cross-sectional area in the regenerating soleus muscle, suggesting a beneficial effect on muscle regeneration without exhibiting the androgenic effects of steroids [19]. The anabolic to androgenic ratio of groups receiving 20-HE and TEAD is very comparable to methandienone (*p* > 0.99). An effect even exceeding methandienone was found in vitro [6], and Chermnykh et al. reported that ecdysterone showed an anabolic effect stronger than that of methandienone [76]. A 10-week intervention study performed on young men investigated the effect of ecdysterone on human sport exercise. A significant increase in muscle mass was observed without an increase in biomarkers for liver or kidney toxicity [7].

It could be concluded that TEAD is a potential anabolic agent containing 20-HE together with other secondary metabolites which previously reported to have anabolic effects and activities that promote muscle health [7,73,74,75,76,77,78]. Moreover, the preparation of a standardized extract of TEAD with potential anabolic activity is of low cost, is time saving and is a simple method compared to the isolation of pure 20-HE [88] Furthermore, TEAD and 20-HE did not affect the cell viability of SkMC with IC_50_ (>100 µg/mL).

## 4. Materials and Methods

### 4.1. Materials

The aerial parts of *A. dimorphostegia* Kar. & Kir were harvested from Wadi Al-Hilali, Arar-Dammam International North Road (50 km from Arar) in March 2018. The position from which the plant was harvested was determined using the global positioning system having coordinates 30°49′47.6″ N 41°14′44.1″ E. The plant collected was identified by Prof. Dr. Ahmed K. Othman, Biology Department, Faculty of Science, Northern Border University, Saudi Arabia. Plant material was then air-dried at room temperature and reduced to a fine powder. A voucher specimen was placed at the herbarium (PHG-P-AM-245) at the Department of Pharmacognosy, Faculty of Pharmacy, Ain Shams University, Egypt. All solvents were of analytical grade, while those used in the preparative HPLC, and UPLC/PDA/ESI/MS assays were of HPLC grade. Methandienone and testosterone enanthate were obtained from Novector labs (East, Maharashtra, India).

### 4.2. Extraction and Fractionation of A. dimorphostegia

Two kilograms of the air-dried *A. dimorphostegia* aerial parts were extracted by maceration in 5 L of absolute ethanol for 24 h at room temperature, followed by filtration. The same process was repeated for four more days until exhaustion. The extracts were collected and dried under reduced pressure at 45 °C yielding 100 g residue. Ten grams of the dried alcohol extract was reserved for further analysis, labeled as TEAD and freeze-dried. The remaining 90 g of the dried extract was suspended in 500 mL distilled water. Fractionation was carried out successively using solvents of increasing polarities: *n*-hexane followed by ethyl acetate. Each fraction was dried completely under reduced pressure to yield *n*-hexane (38 g) and ethyl acetate (14 g) residues, respectively. The residues were then freeze-dried for further investigations.

### 4.3. Isolation of 20-HE from EFAD

EFAD was subjected to column chromatography. EFAD (4 g) was dissolved in 20 mL of distilled water and chromatographed over a 160 g Diaion HP-20 (Merck, Germany) column (2.5 × 100 cm). Fractions were eluted using water and methanol (H_2_O: MeOH): 0–100% step-gradient with an increment of 20% methanol every 1000 mL. The column was flushed with 200 mL of 100% methanol for the last three fractions. Elution was monitored using TLC and (DCM: MeOH) (9:1) as the mobile phase. Eluents from 0–40%, 60–80% and 100% methanol were pooled into fractions 1, 2 and 3, respectively. Fraction 2 (60–80%) was further refined using column chromatography. The fraction weighing 1 g was dissolved in 5 mL DCM and chromatographed using 50 g Silica gel 60 (Merck, Germany) column (1.5 × 100 cm). DCM and MeOH were used as eluents with a ratio of 9.5:0.5. Elution was monitored using TLC in a solvent system of DCM and MeOH (9:1). Fractions 101–153 showed a single green band after spraying with p-anisaldehyde/H_2_SO_4_, suggesting the presence of triterpenoid skeleton. Thus, it was pooled and dried under reduced pressure at 45 °C, yielding 55 mg white crystalline solid. 

### 4.4. NMR Spectrometrical Analysis of 20-HE

NMR spectra were conducted using a Bruker Avance 400 MHz NMR spectrometer using DMSO-*d*_6_ as a solvent. One-dimensional ^1^H and ^13^C spectra were obtained using standard pulse sequences and parameters. ^1^H chemical shifts (δ) were measured in ppm, relative to TMS and ^13^C NMR chemical shifts to DMSO-*d*_6_, and were converted to the TMS scale by adding 39.49. 

### 4.5. LC/ESI/MS Spectrometrical Analysis of 20-HE

LC-ESI-MS spectrometrical analysis of 20-HE Waters ™ ACQUITY Xevo ™ TQD system (Waters Corp., Milford, MA, USA). A C_18_ column with dimensions of 2.1 mm × 50 mm, 1.7 μm (Waters, Ireland) was used. 20-HE was dissolved in methanol at a concentration of 100 μg/mL, then filtered using a 0.2 micropore filter. A gradient elution program was conducted using eluent A (0.1% formic acid in water) and eluent B (0.1% formic acid in methanol). The total run time was 32 min and a flow rate was kept at 0.2 mL/min. For mass spectrometric detection, a XEVO TQD triple quadrupole mass spectrometer equipped with an electro-spray ionization (ESI) source (Waters Corp., Milford, MA, USA) was used. Mass spectra were detected in the ESI between *m*/*z* 100–1000. The peaks and spectra were processed using the Maslynx 4.1 software (Waters, Milford, MA, USA). 

### 4.6. UPLC-PDA-ESI-MS/MS Analysis of TEAD

The profiling of TEAD was performed on a Waters™ ACQUITY Xevo ™ TQD system (Waters Corp., Milford, MA, USA). A C_18_ column with dimensions of 100 mm × 2.1 mm (p.s., 1.7 µm) (Waters, Ireland) was used. The sample was dissolved in methanol at a concentration of 1 mg/mL and was then filtered using a 0.2 µm micropore filter. The instrument settings and the gradient elution program were conducted using the method described earlier by El Zahar et al. using eluent A (0.1% formic acid in water) and eluent B (0.1% formic acid in acetonitrile) [89]. The flow rate was kept at 200 μL/min with an injection volume of 10 µL.

### 4.7. Standardisation of TEAD Using UPLC Analysis

Chromatographic analysis of alcohol extract was performed using an UPLC system Thermo Fisher UHPLC Dionex Ultimate 3000 (Germering, Germany). The system is equipped with a (ISO-3100SD) pump, (WPS 3000 SL) autosampler, (TCC-3000SD) column thermostat and (DAD-3000 RS) diode array detector (Germering, Germany). The software used was Chromeleon 6.8 (Germering; Germany). A column of Hypersil Gold was used with a 5 μm particle size and dimensions of 250 × 4.6 mm. The solvents used were 1% orthophosphoric acid in water (A) and acetonitrile (B). The separation was performed for a total of 70 min using gradient elution from 10% to 75% B in A for the first 35 min at a flow rate 0.5 mL/min, and then increased to 0.8 mL/min for the whole run. Then, the elution went from 75% to 100% B in A for 15 min and was kept isocratic for another 15 min. For the next 15 min, the elution was from 100% to 40% B in A and for the last 10 min kept isocratic from 10% B in A. UV detection was at 213 nm, column temperature at 30 °C and the injection volume was 20 μL. The isolated 20-HE (1 mg) was dissolved in 1 mL methanol, serial dilutions were performed using five concentrations (1, 10, 20, 40 and 80 μg/mL) and a calibration curve was generated using the same method [90].

### 4.8. In Silico Modelling of Steroid Receptor Binding

The molecular docking experiments were carried out using protein crystal structures obtained from the Protein Data Bank (PDB) [91]. The structures used were 3OLL, 3UUD and 2AM9 for ERβ, ERα and AR, respectively [84,85,86]. The PDB structures were prepared using the Dock Prep protocol embedded in Chimera [92]. The docked ligands were prepared using the same protocol. Molecular docking was then carried out using the AutoDock Vina extension in Chimera [93]. A docking grid was generated with dimensions 25 × 25 × 25 Å for all the docking experiments. The position of the docking grid center is (−26, 25, −12), (20, 7, 5) and (27, 3, 5) for ERβ, ERα and AR, respectively, using an (x, y, z) format. A default exhaustiveness value of 8 and an energy range of 3 kcal/mol were used for generating binding poses. The resulting poses were evaluated using Maestro Visualizer (Maestro, Schrödinger, LLC, New York, NY, USA, 2021) and figures and graphics were generated using the same software. To validate the docking protocol, the co-crystallized ligands were re-docked using the same docking parameters. The generated docking poses were identical to the co-crystallized poses for ERβ, ERα and AR with RMSD values of 0.508, 0.643 and 0.751, respectively. This indicates a valid docking protocol.

### 4.9. Evaluation of the Cytotoxic Activity of 20-HE and TEAD (SRB-U Assay)

The human skeletal muscle cell line was cultivated as adherent cultures on Petri dishes and kept at 37 °C. The SRB-U assay was used to assess cell viability [94,95] Three independent experiments with three internal replicates were performed. All data were blank adjusted prior to further interpretation.

### 4.10. In Vivo Anabolic Study

#### 4.10.1. Animals

Handling of animals and experimental design were approved and conducted according to the guidelines of the Research Ethics Committee of the Faculty of Pharmacy, British University in Egypt, Cairo. (Approval No. EX-2217). Twenty-five immature (21 days old) male Wistar rats weighing approximately 60 g were obtained from Nile Co. for Pharmaceutical and Chemical Industries, Cairo, Egypt. Animals were housed in a temperature-controlled environment at 25 °C with alternating 12 h light and dark cycles. They were kept on a standard diet and water ad libitum.

#### 4.10.2. Orchidectomy

Orchidectomy was performed using the technique described earlier [1]. General anesthesia was performed with the association of Ketamine (120 mg/kg) and Xylazine (16 mg/kg) administered in the same syringe as 1.0 mL. The rat was restrained in dorsal recumbency, and the surrounding scrotal area was prepared for aseptic surgery. Using a scalpel blade, a 1 cm incision was made through the skin ventrally on both sides of the scrotum. The testis was then explored and retracted to expose a section of the vas deferens and the spermatic cord’s vasculature, ligated, then tied in an overhand knot. The cord was then excised, and the process was repeated on the other side. Finally, muscle and skin closure was performed with two stitches using synthetic absorbable sutures and then observed for a few hours for signs of hemorrhage. Rats were then left to recover for one week; two rats died at days 2 and 5.

#### 4.10.3. Experimental Design

**Figure 16:**
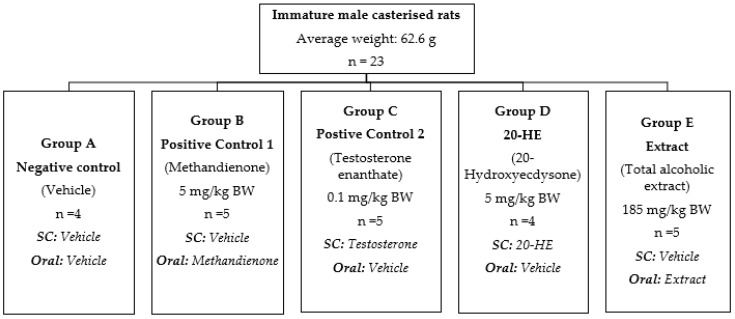
The scheme of experimental design for the study of the effect of 20-HE and total alcoholic extract on muscle fibers and secondary sex organs.

Twenty-three rats with an average weight of 62.25 ± 10.26 g were randomly divided into five groups and treated with test compounds by gavage and subcutaneous (SC) injection (Figure 16). Group A received only the vehicle (20% ethanol and 80% corn oil). Groups B to E received methandienone, testosterone enanthate, 20-HE and TEAD with concentrations of 5 mg/kg, 0.1 mg/kg, 5 mg/kg and 185 mg/kg, respectively, each diluted in a solution of 20% ethanol and 80% corn oil. The rats were administered once daily with the respective substances for ten consecutive days.

#### 4.10.4. Sample Collection

On day 10, rats were killed by cervical dislocation. The seminal vesicles (S. vesicles), ventral prostate and levator ani were dissected out and weighed to the nearest 0.1 mg. The seminal vesicles were squeezed before weighing to remove any fluids. The soleus and gastrocnemius muscles were then dissected together as a bundle (muscle bundle) from the right and left hind limb of rats, weighed immediately and then stored in 10% neutral buffered formalin for 72 h for further investigation.

#### 4.10.5. Histopathological Examination and Determination of Muscle Fiber Cross-Sectional Area

Samples were trimmed and processed in serial grades of ethanol, cleared in Xylene, synthetic paraffin wax infiltration and embedded into Paraplast tissue embedding media. Then, 4 μm thick cross sections were cut by rotatory microtome. The sections were stained with Harris hematoxylin and eosin as a general tissue examination staining method. All methods of tissue samples processing and staining were as outlined before [96]. Nine random non-overlapping fields per sample tissue section were analyzed for determination of muscle fibers cross sectional area. All data and micrographs were obtained by using a Full HD microscopic camera operated by the Leica application module for tissue sections analysis (Leica Microsystems GmbH, Germany, Berlin).

#### 4.10.6. Statistical Analysis

All statistical calculations and generation of graphs and the cumulative data of weight gain in rats, secondary sex organs and muscle fibers, as well as the muscle fiber’s cross-sectional area, were compared by using the t-test for unpaired samples and one-way ANOVA analysis followed by Tukey–Kramer as a post hoc test. All tests were performed using GraphPad Prism version 7.00. The data are expressed as means ± SD.

## 5. Conclusions

The *Atriplex dimorphostegia* total ethanol extract proved anabolic potency. This was represented by the increase in net muscle mass of gastrocnemius and soleus muscle, as well as an increase in the cross-sectional area of muscle fibers. Moreover, both 20-HE and TEAD exhibited a low androgenic effect on sex organs without increasing the weights of prostate and seminal vesicles of rats after administration for ten consecutive days. These findings indicate that *A. dimorphostegia* is a promising candidate for use as an anabolic agent and warrants further clinical investigation. Moreover, future study of the pharmacokinetics of TEAD and 2-HE in rats, as well as the lifespan, biodistribution, and muscle tissue retention, will be conducted to evaluate the biological effects of indicated doses.

## Figures and Tables

**Table 1:** ^1^H- and ^13^C-NMR assignment of 20-HE.

Position	δ _ H _	δ _ C _
1α	1.97	38.89
1β	1.48	
2	3.8	67.22
3	4.11	67.02
4α	1.77	30.21
4β	1.61	
5	2.39	50.52
6	-	203.8
7	5.82	120.85
8	-	166.09
9	3.13	38.09
10	-	39.73
11α	1.81	22.79
11β	1.61	
12α	2.10	30.11
12β	1.90	
13	-	49.12
14	-	83.55
15α	2.17	29.28
15β	1.58	
16α	1.81	23.69
16β	1.97	
17	2.51	49.12
18	0.89	17.54
19	1.05	24.20
20	-	76.34
21	1.22	21.27
22	3.49	76.76
23α	1.73	24.91
23β	1.32	
24α	1.83	41.65
24β	1.46	
25	-	69.49
26	1.22	26.44
27	1.25	28.76

**Table 2:** Peak assignment using UPLC-PDA-ESI-MS/MS of the active metabolites detected in *A. dimorphostegia* L.

Peak #	Retention Time (min)	Identified Compound	UV-Vis (λmax)	[M−H^]−^ (*m*/*z*)	[M+H]^+^ (*m*/*z*)	Fragment Ions (*m*/*z*)	Peak Area (%)	Occurrence	Reference
1	0.205	Caffeoyl hexose-deoxyhexoside	232, 318	487.01		307, 179	0.02	*-*	[42]
2	0.306	Chlorogenic acid derivative	220, 325	451.12		353, 191	0.95	*A. mollis*	[43,44]
3	0.37	Isoorientin	238, 349	448.2		357, 327	0.89	*A. halimus*	[45,46]
4	0.42	Apigenin dihexoside	242, 334	593.4		269	1.29	*A. halimus*	[45,47]
5	0.56	Liquiritin	239, 335	417.01		255	0.78	*-*	[44]
6	0.65	Ferulic acid	235	193.1		178, 161, 134	0.75	*A. mollis*	[43,48]
7	0.78	Kaempferol deoxyhexoside	245, 348	431.04		285	0.52	*A. lentiformis*	[49]
8	0.795	Dihydroxy benzoic acid	247, 314	153.0		108	2.25	*A. hortensis*	[50]
9	0.92	Caffeic acid	233	179.01		135, 107	0.99	*A. lindelyi*	[26,51]
10	0.95	Caffeoyl hexose-deoxyhexoside isomer	233, 322	487.08		308, 179	0.36	*-*	[42]
11	1.01	Quercetin pentosyl hexoside	235, 355	595.2		463, 301	0.99	*A. lindelyi*	[26]
12	1.021	Rosmarinic acid hexoside	232, 318	521.1		359, 197	0.45	*-*	[52]
13	1.138	Retusin methyl ether	230, 282	297.3		282, 239, 211	0.69	*-*	[53]
14	1.2	Caffeic acid derivative	244, 327	295		135, 179	1.06	*-*	[54]
15	1.3	Ferulic acid ester derivative	241, 327	309.53		192, 177, 115	0.63	*-*	[54]
16	1.5	Tetrahydroxyflavan (Afzelechin)	242, 323	273.1		255, 179	0.76	*-*	[48]
17	2.12	Kaempferol	250, 370	285.10		153	0.65	*A. halimus*	[49,55]
18	2.2	Isorhamnetin	244, 368	315.2		301,271, 151	0.34	*A. lindelyi*	[26,56]
19	2.23	Myricetin	243, 372	317.1		179, 151	0.42	*A. halimus*	[56,57]
20	2.24	Quercetin	238, 374	301.0		179, 151	0.09	*A. halimus* *A. lindelyi*	[26,57]
21	2.25	Dihydroxybenzoyl hexose	nd	316.1		153,108	1.08	*A. lindelyi*	[26]
22	3.58	Coumaroyl hydroxy-palmitic acid	223	418.5		163, 145, 119,	2.50	*-*	[48]
23	4.23	Quercetin-galloyl-pentoside	236, 354	585		301, 179, 153	2.80	*-*	[58]
24	4.52	Coumaroyl-hexose	228, 315	325.65		163	0.07	*-*	[59]
25	4.52	Kaempferol–dideoxyhexoside (Kaempferitrin)	246, 354	577		285	1.33		[49]
26	4.71	Dicaffeoyl-spermidine	232, 309	468.2		332, 135	0.98	*-*	[51]
27	4.75	Syringetin rutinoside	222, 312	579.2		345	1.02	*A. halimus*	[20,52]
28	4.79	Acetylated kaempferol deoxyhexosyl hexoside	243, 350	635.2		299, 284	3.59	*-*	[52]
29	4.92	Kaempferol deoxyhexosyl hexoside	262, 355	593.1		447, 285	0.55	*A. lentiformis*	[49]
30	5.3	Isorhamnetin hexoside	268, 355	477		315, 271	1.07	*-*	[60]
31	5.95	Quercetin deoxyhexoside	225, 358	447		301,179	1.20	*A. centralasiatica*	[61,62]
32	6.07	Apigenin-C-hexoside	269, 334	431.01		311, 341	0.77	*-*	[61,63]
33	6.52	Daucosterol (Sitogluside)	255, 278		577	413, 369	1.96	*A. centralasiatica*	[64]
34	6.52	Isorhamnetin deoxhexosyl hexoside	268, 356	623.3		315, 301, 179, 151	2.52	*A. halimus*	[20,60]
35	6.78	Kaempferol glucuronide	247, 354	513.1		461, 285, 175,135	0.08	*-*	[65]
36	6.92	Sinapic acid hexoside	228	385		223	1.04	*-*	[66]
37	7.3	Tamerexetin deoxyhexosyl hexoside	250, 368	623.2		325, 315	1.71	*A. halimus*	[20]
38	7.5	Apigenin-C-hexoside	270, 336	431		353, 341, 311, 269	0.59	*-*	[61,63]
39	7.9	Caffeoyl-coumaroyl spermidine	223	452.4		332, 316, 306, 289, 135	2.90	*-*	[51]
40	8.75	β-Sitosterol			415.5	397, 175, 257	5.66	*A. stocksii*	[64,67]
41	9.01	Caffeoyl pinoresinol	232, 309	519.5		357, 151	0.85	*-*	[68]
42	9.23	Tetrahydroxyflavan (Afzelechin)	242, 323	273.1		255, 179	0.92	*-*	[48]
43	9.85	Kaempferol	250, 370	285.10		153	1.60	*A. lentiformis*	[49]
44	10.01	Portulasoid butoxyseptanoside	265	/237		253	1.52	*A. portulacoides*	[36]
45	10.2	Isorhamnetin	244, 368	315.2		301, 271, 151	1.71	*A. halimus*	[56,57]
46	11.05	Myricetin	243, 372	317.1		179, 151	1.91	*A. halimus*	[56,57]
47	11.51	Quercetin	238, 374	301.0		179, 151	2.02	*A. lindelyi*	[26]
48	11.59	Tetrahydroxyflavan (Afzelechin)	242, 323	273.1		255, 179	0.73	*-*	[48]
49	11.84	Ursolic acid	226		457	407, 391	1.68	*A. stocksii*	[67,69]
50	12.05	β/α-amyrin	255, 270		427.2	218, 203, 189	2.08	*A. stocksii*	[67,70]
51	12.5	20-hydroxyecdysone	248		481	445, 427, 409, 391	11.91	*A. portulacoides*	[36,71]
52	12.8	β/α-amyrin	270		427.2	218, 203, 189	1.25	*A. stocksii*	[67,70]
53	13.2	Lupeol	265		427.5	409, 397	0.52	*A. lindelyi*	[72,73]
54	13.55	Stigmasterol	285		413.2	395, 411	3.02	*A. lindelyi*	[73,74]
55	14.57	Oleonolic acid	260		457	439, 411, 393	1.36	*A. stocksii*	[67,75]
56	14.89	Septanoecdysone	255		657.2	521, 439	0.22	*A. portulacoides*	[36]

## Data Availability

The data that support the findings of this study are openly available from the corresponding authors upon reasonable request.
